# Exploring the role of COX-2 in Alzheimer's disease: Potential therapeutic implications of COX-2 inhibitors

**DOI:** 10.1016/j.jsps.2023.101729

**Published:** 2023-08-07

**Authors:** Nathalie Moussa, Ninar Dayoub

**Affiliations:** a Department of Pharmaceutical Chemistry and Drug Control, University of Manara, Latakia, Syria; b Faculty of Pharmacy, University of AL Andalus for Medical Science, Tartus, Syria

**Keywords:** Alzheimer's disease, COX-2, NSAIDs, Drug discovery, CDK5, Tau phosphorylation

## Abstract

This review highlights the potential role of cyclooxygenase-2 enzyme (COX-2) in the pathogenesis of Alzheimer's disease (AD) and the potential therapeutic use of non-steroidal anti-inflammatory drugs (NSAIDs) in the management of AD. In addition to COX-2 enzymes role in inflammation, the formation of amyloid plaques and neurofibrillary tangles in the brain, the review emphasizes that COXs-2 have a crucial role in normal synaptic activity and plasticity, and have a relationship with acetylcholine, tau protein, and beta-amyloid (Aβ) which are the main causes of Alzheimer's disease. Furthermore, the review points out that COX-2 enzymes have a relationship with kinase enzymes, including Cyclin Dependent Kinase 5 (CDK5) and Glycogen Synthase Kinase 3β (GSK3β), which are known to play a role in tau phosphorylation and are strongly associated with Alzheimer's disease. Therefore, the use of drugs like NSAIDs may be a hopeful approach for managing AD.

However, results from studies examining the effectiveness of NSAIDs in treating AD have been mixed and further research is needed to fully understand the mechanisms by which COX-2 and NSAIDs may be involved in the development and progression of AD and to identify new therapeutic strategies.

## 1 Introduction

Alzheimer's Disease (AD) is a progressive and debilitating neurodegenerative disease that affects millions of people worldwide. It is characterized by a gradual deterioration of cognitive function, leading to memory loss, confusion, and difficulty in performing daily tasks. AD usually develops in people over the age of 65, but it can also occur in younger individuals (https://www.nia.nih.gov/health/alzheimers-disease-fact-sheet). The disease is caused by the accumulation of abnormal proteins, such as amyloid plaques and tau tangles, in the brain which leads to the loss of connections between nerve cells and brain tissue. This process is irreversible and leads to a decline in cognitive function, ultimately resulting in dementia. AD is one of the most common causes of dementia and it is a significant public health concern (Breijyeh and Karaman, 2020, McGirr et al., 2020, Scheltens et al., 2021).

Alzheimer's disease is a very complex disease with multiple causes and hypotheses. Aβ plaques and tau tangles are considered to be key pathological factors of AD. Aβ plaques are composed of aggregated amyloid-β protein, which is believed to play a role in the death of neurons and synapses. Tau tangles, on the other hand, are composed of hyperphosphorylated tau protein, which disrupts the normal function of microtubules in neurons and causes neuronal loss. In addition to these features, AD is also associated with inflammation, synaptic and neuronal loss, loss of cholinergic neurons, and other factors (Xuereb et al., 1991, Gerakis and Hetz, 2018, Gendron and Petrucelli, 2009, Lu et al., 1999, Iqbal et al., 2010).

Tau is a microtubule-associated protein that plays an important role in the stability and modulation of internal microtubules in the brain. In healthy individuals, the phosphorylation of tau protein is regulated by a balance between the activities of tau kinases and tau phosphatases. However, in the brains of people with AD, tau becomes hyperphosphorylated, which means that the protein detaches from microtubules and clings to other tau molecules forming fibers that accumulate as neurofibrillary tangles (NFTs) (Umeda et al., 2014, Hurtado et al., 2010, Serrano-Pozo et al., 2011).

The formation of NFTs is thought to be caused by an imbalance between kinases and phosphatases in the brain. Kinases are enzymes that add a phosphate group to a protein, while phosphatases are enzymes that remove a phosphate group from a protein. In AD, there is an overactivity of kinases, particularly GSK3ß and CDK5, which leads to the abnormal hyperphosphorylation of tau protein and the formation of NFTs (Wainaina et al., 2014, Miao et al., 2019, Noble et al., 2003).

GSK3ß and CDK5 are important tau protein kinases that are known to be involved in the pathogenesis of AD. GSK3ß is a protein kinase that plays an important role in several aspects of neuronal function and is known to be involved in the formation of NFTs. CDK5, on the other hand, is a protein kinase that plays a critical role in normal development of the central nervous system and is also known to contribute to the formation of NFTs (Flaherty et al., 2000, Braithwaite et al., 2012, Hossain et al., 2019).

Therefore, the inhibition of these enzymes is an auspicious therapeutic plan for managing AD by reducing the formation of NFTs and slowing the progression of the disease.

Recent research has focused on targeting tau accumulation because of the strong association with symptoms of memory loss. Studies have shown that tau tangles are closely linked to cognitive decline and dementia in AD, and it is believed that reducing tau accumulation could help slow the progression of the disease and improve symptoms (Ossenkoppele et al., 2022, Takashima, 2009). This is an active area of research, and many researchers are working to develop new therapies that target tau accumulation and its underlying mechanisms (Du et al., 2018, Maass et al., 2018).

It's worth noting that AD is a multifactorial disease and targeting one specific pathway or protein may not be the solution for all patients. Therefore, research should continue to explore other potential targets and to develop a combination of therapies that can target multiple pathways (Jack et al., 2018, Herrmann et al., 2011).

COX-2, a key enzyme in the production of proinflammatory molecules, has been found to play a significant role in the pathogenesis of Alzheimer's disease (AD). Studies have shown that COX-2 overexpression leads to tau hyperphosphorylation and the formation of neurofibrillary tangles (Guan et al., 2019, Guan and Wang, 2019, Zhang and Jiang, 2015). Additionally, research has revealed that COX-2 is involved in the regulation of kinases and phosphatases, which are essential for maintaining the balance between tau phosphorylation and dephosphorylation (Arnaud et al., 2006, Duan et al., 2014). This imbalance between kinases and phosphatases is thought to be a major contributor to tau hyperphosphorylation and the formation of NFTs (Liu et al., 2015, Rahman et al., 2005, Kumar et al., 2014).

Furthermore, studies have revealed a link between neuroinflammation and AD, with activated microglia producing proinflammatory molecules that contribute to the progression of the disease (Cao et al., 2019, Onyango et al., 2021, Webers et al., 2020).

Given the strong association between COX-2, tau phosphorylation, and neuroinflammation in AD, non-selective and COX-2 selective inhibitors are being investigated as potential therapeutic agents for managing AD. However, some studies have found that these drugs may not be effective in slowing the progression of the disease, raising questions about the potential explanations for their failure and the timing of treatment. Nevertheless, further research is needed to fully understand the potential of these drugs as therapeutic agents for managing AD (Jope et al., 2007, Wilkaniec et al., 2018).

In this review, we aim to clarify the role of cox-2 enzyme in some Alzheimer's hypotheses and the beneficial effects of using non-selective and COX-2 selective inhibitors as promising therapeutic agents to slow down the progression of AD or mild cognitive dementia.

## 2 Cyclooxygenase enzymes

Prostaglandin (PG) G/H synthase or most commonly referred to as Cyclooxygenase (COX) enzymes are a group of enzymes that convert arachidonic acid to eicosanoids which are mediators that serve important functions in normal physiology and also have a role in pathological conditions (Ali et al., 2023, Wang et al., 2021b, Wang et al., 2021a). This process begins with the release of a poly unsaturated fatty acid processing 20 carbon atoms called arachidonic acid (AA) (Tallima and El Ridi, 2018) present in the phospholipids of cell membrane by the action of the enzyme phospholipase A2 (PLA2), AA is converted by the COX enzymes to PGG2 and PGH2 (Wang et al., 2021b, Wang et al., 2021a), PGH2 is converted to different prostanoids species which are a subclass of eicosanoids such as prostacyclin (PGI2), prostaglandin D2 (PGD2), prostaglandin E2 (PGE2), prostaglandin F2α (PGF2α) and thromboxane A2 (TXA2) (Mitchell and Kirkby, 2019), COX enzymes comprise COX-1 and COX-2 isoforms, COX-1 is the constitutive form of the enzyme, is expressed under normal physiological conditions and is found in such tissues as the kidney, on the other hand, COX-2 is the inducible form of the enzyme, is expressed in response to inflammatory stimuli, while COX-3 which is made from the COX-1 gene has been detected in human cerebral cortex (Gunaydin and Bilge, 2018), as a result, COX-2 is considered as an important therapeutic target to reduce inflammation in various diseases, including Alzheimer's Disease (AD) (Biringer et al., 2019; Liu et al., 2022, Tyagi et al., 2020). Non-steroidal anti-inflammatory drugs (NSAIDs) can block incipient inflammation-driven AD pathogenesis by inhibiting the activity of COX enzymes, leading to a reduction in the secretion of prostaglandins and inflammation (Maccioni et al., 2020, Woodling et al., 2016).

Cyclooxygenase-2 (COX-2) enzyme inhibitors have not eliminated the necessity for developed drugs in both the fields of synthesis and drug design methods. Many series of novel molecules have been reported as selective COX-2 inhibitors and still being reported to these days. The researchers have always stated that COX-2 inhibitors are needed not only in the non-steroidal anti-inflammatory drugs (NSAIDs) area but also in other therapeutic applications, including prevention of cancer and Alzheimer's disease (AD) (Abdellatif et al., 2022, Ayman et al., 2023, Chen et al., 2023, Kumar et al., 2023, Moussa et al., 2021).

There are a number of reasons why researchers may be interested in synthesizing new COX-2 inhibitors for AD. First, as previously discussed, COX-2 has been implicated in the pathogenesis of AD and has been shown to be upregulated in the brains of AD patients (Yang, 2019, Tyagi et al., 2020). Second, non-steroidal anti-inflammatory drugs (NSAIDs), which are known inhibitors of COX enzymes, have been shown to have beneficial effects in the management of AD (Villarejo-Galende et al., 2020; Meyer et al., 2019, Fang et al., 2022, Benito-León et al., 2019). Third, the failure of some COX-2 inhibitors in clinical trials for AD may be due to their selectivity for COX-2 over COX-1, and so, new non-selective COX inhibitors or COX-2 inhibitors with different mechanisms of action may be more effective (Scharf et al., 1999, Imbimbo, 2009). Finally, new COX-2 inhibitors may have improved pharmacological properties, such as improved solubility or bioavailability, which would increase their efficacy and safety. Therefore, research in this area aims to improve the current COX-2 inhibitors, or develop new compounds that interact with different mechanisms (McGeer et al., 1996). Overall, COX-2 inhibitors reduce inflammation and prevent the progression of AD.

## 3 Role of neuronal COX expression in Alzheimer's disease

The potential role of cyclooxygenase enzymes gained one of the highest scientific interests in the research field since it was suggested that COX may exist at a crossroad of neuroinflammation and neurodegeneration. COX-2 is generally considered inducible and is significantly involved in the brain inflammation (Zarghi and Arfaei, 2011, Jung et al., 2019). As it has been shown that COX-2 is induced by variety of inflammatory mediators including cytokines, IL-1, IL-2 and TNF-α (Tyagi et al., 2020, Belvisi et al., 1997). COX-2 is also constitutively expressed in distinct regions of the brain, including the neocortex (Hoozemans et al., 2008) and the hippocampus which is divided into dentate gyrus, CA1, CA2, CA3, and subiculum (Kendall et al., 1991). The dentate gyrus plays an essential role in the formation of hippocampal memory (Hainmueller and Bartos, 2020). the results of the present study suggest that COX–2 serves an important role in synaptic plasticity in the dentate gyrus and changes in the levels of its constitutive expression are associated with the hippocampal dentate gyrus postnatal development (Jung et al., 2019). The inducible form of COX (COX-2) may be involved in neuroinflammatory and neurodegenerative conditions. COX-2 has been found to increase the production of proinflammatory molecules, such as interleukin-1beta (IL-1beta) and tumor necrosis factor-alpha (TNF-alpha), which can contribute to the development of neuroinflammation and further neuronal damage (Rubio-Perez and Morillas-Ruiz, 2012). Therefore, a lot of work has been dedicated to studying the role of this enzyme in neurodegenerative diseases such as Alzheimer's (López and Ballaz, 2020).

A study has been showed that the activity of COX-2 in the hippocampus serves an important role in learning and memory functions (Minghetti, 2004). Some studies found that overexpression of COX-2 correlated with impairment of spatial memory, which is one of the main symptoms of Alzheimer's disease (Woodling et al., 2016, Minghetti, 2007).

**Fig. 1:**
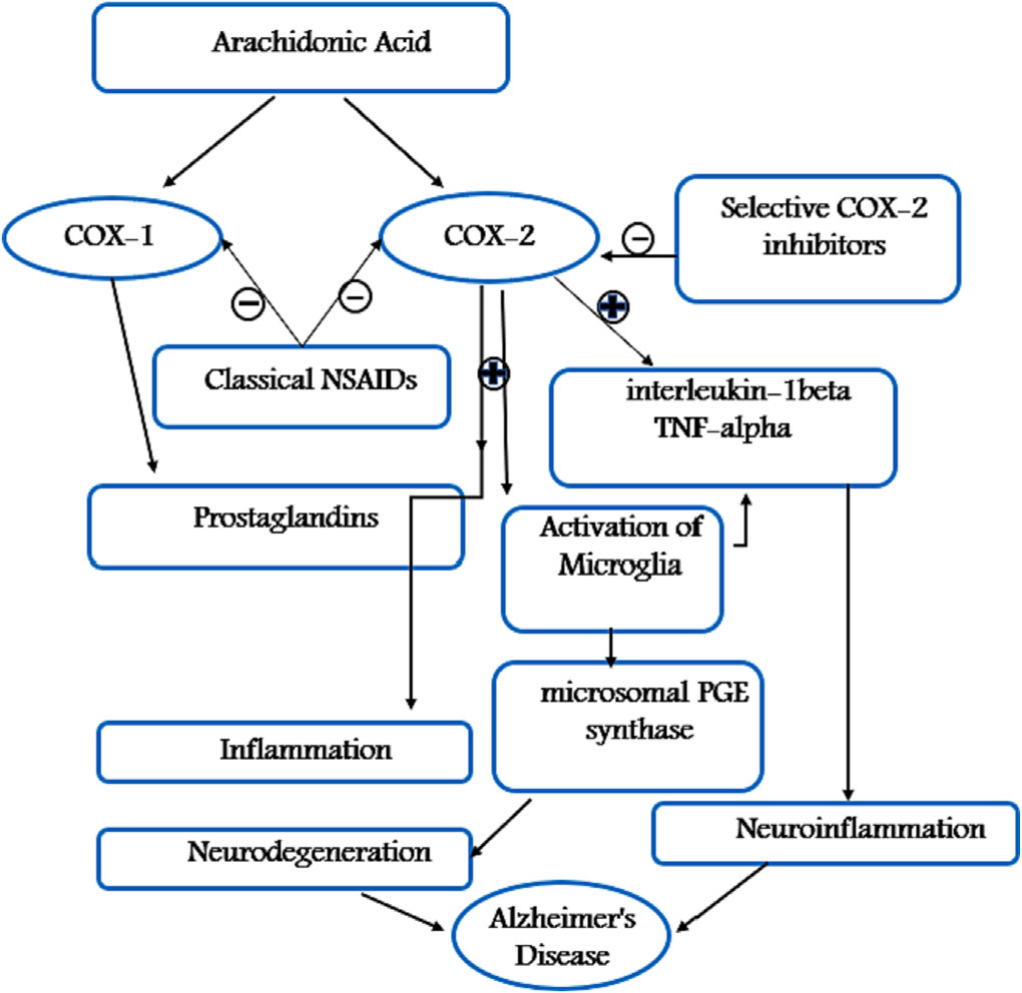
Role of COX-2 enzyme in neuroinflammation and neurodegeneration associated with Alzheimer's Disease.

According to many in Virto and in vivo studies, COX-2 is expressed under normal physiological conditions such as synaptic activity and plasticity (Tyagi et al., 2020) which controls how two neurons make new communications with each other (Rendeiro and Rhodes, 2021). The induction of neuronal COX-2 is increased upon synaptic activity, it is also induced in glial cells during inflammatory process and is expressed under normal conditions in hippocampal neurons including tri-synaptic loop, notably in layers II/III of entorhinal cortex, the dentate granule cell layer, and CA3–CA1 neurons (brain regions affected at early stages of Alzheimer’s disease) (Woodling et al., 2016, Kawaguchi et al., 2005). Many studies reported that COX-2 expression is induced in both astroglia (López and Ballaz, 2020) and microglia which have an essential role in number of functions such as homeostasis and controlling neuronal functions (Tyagi et al., 2020, Leng and Edison, 2021). Therefore, it was suggested that COX-2 expression leads to an activation of microglia or astroglia which correlates with disease development (Guan and Wang, 2019, Wang et al., 2014). When microglial cells are activated, the secretion of pro-inflammatory cytokines including TNF-α and IL-1β, increases leading to a neuroinflammatory response (López and Ballaz, 2020). The activation of microglial cells is also associated with the release of microsomal PGE synthase (mPGES)-1 (Nagano et al., 2021) which is an inducible enzyme involved in the synthesis of prostaglandins E2 (PGE_2_) leading to neurodegenerative diseases (Chini et al., 2020, Iwata et al., 2021). Fig. 1 illustrates the relationship between COX-2 and neuroinflammation.

The expression of COX varies according to the different stages of AD, during the early phase of the disease, neuronal COX-2 expression is increased (Tyagi et al., 2020) causing aberrant hippocampal synaptic activity and neuronal death (Hoozemans et al., 2008, Yermakova, 2001). Other studies confirmed that high levels of COX-2 in some hippocampal neurons are correlated with the development of AD (Ho et al., 2001), but for the later stages of AD, immunostaining of post-mortem AD brains revealed that COX-2 positive neurons decrease as the disease progresses (Biringer, 2019, López and Ballaz, 2020). This suggests that COX-2 may play a role in the early stages of AD and could also indicate that COX-2 inhibitors may be more effective in the early stages of AD, rather than in later stages. Additionally, the decrease in COX-2 positive neurons in later stages of AD could also suggest that other mechanisms and enzymes may be involved in the progression of the disease (Woodling et al., 2016, Hoozemans et al., 2008). However, more research is needed to study the role of COX-2 in the different stages of AD, and to determine the optimal timing for treatment with COX-2 inhibitors.

In addition to COX-2 involvement in AD, there is strong evidence from preclinical and postmortem studies to indicate that COX-1 also has a role in AD (Cui et al., 2004), in both early and later stages of AD, COX-1 Immunostaining obtained from both AD and control brain tissue found that microglia express COX-1 in AD and control brains (Biringer, 2019, Yermakova et al., 1999). COX-1 has a prominent role in the neuroinflammatory cascade because of the observed increase of COX-1 activity in microglial cells (Shukuri et al., 2016). Selective COX-1 inhibition reduces neuroinflammation and improves cognitive function in mice (Choi et al., 2013). COX-3 may also be involved in AD. COX-3 is expressed in the same affected brain areas of Alzheimer's (Cuo et al., 2004). These findings are indicative of the potential contributions of cyclooxygenase enzymes to neuroinflammation-induced cognitive impairment (Choi et al., 2013).

It is worth noting that COX-2 inhibitors are not without potential side effects, as some studies have shown that long-term use of COX-2 inhibitors may increase the risk of cardiovascular events such as heart attack and stroke (Das, 2005, Patrono, 2016). Also, COX-2 inhibitors have been shown to have a negative impact on bone health, leading to an increased risk of fractures. However, these risks must be balanced with the potential benefits of COX-2 inhibitors in managing neurodegenerative diseases such as AD. Moreover, there is a need for more research in this field to examine the specific mechanisms of action of COX-2 inhibitors and other NSAIDs in AD, as well as to identify the most appropriate patients for treatment with these drugs. Additionally, we need to develop new COX-2 inhibitors with fewer side effects and to investigate the potential benefits of targeting other COX enzymes such as COX-1 and COX-3 in the management of AD.

## 4 COX-2 and Nuclear factor Kappa B (NF-kB)

**Fig. 2:**
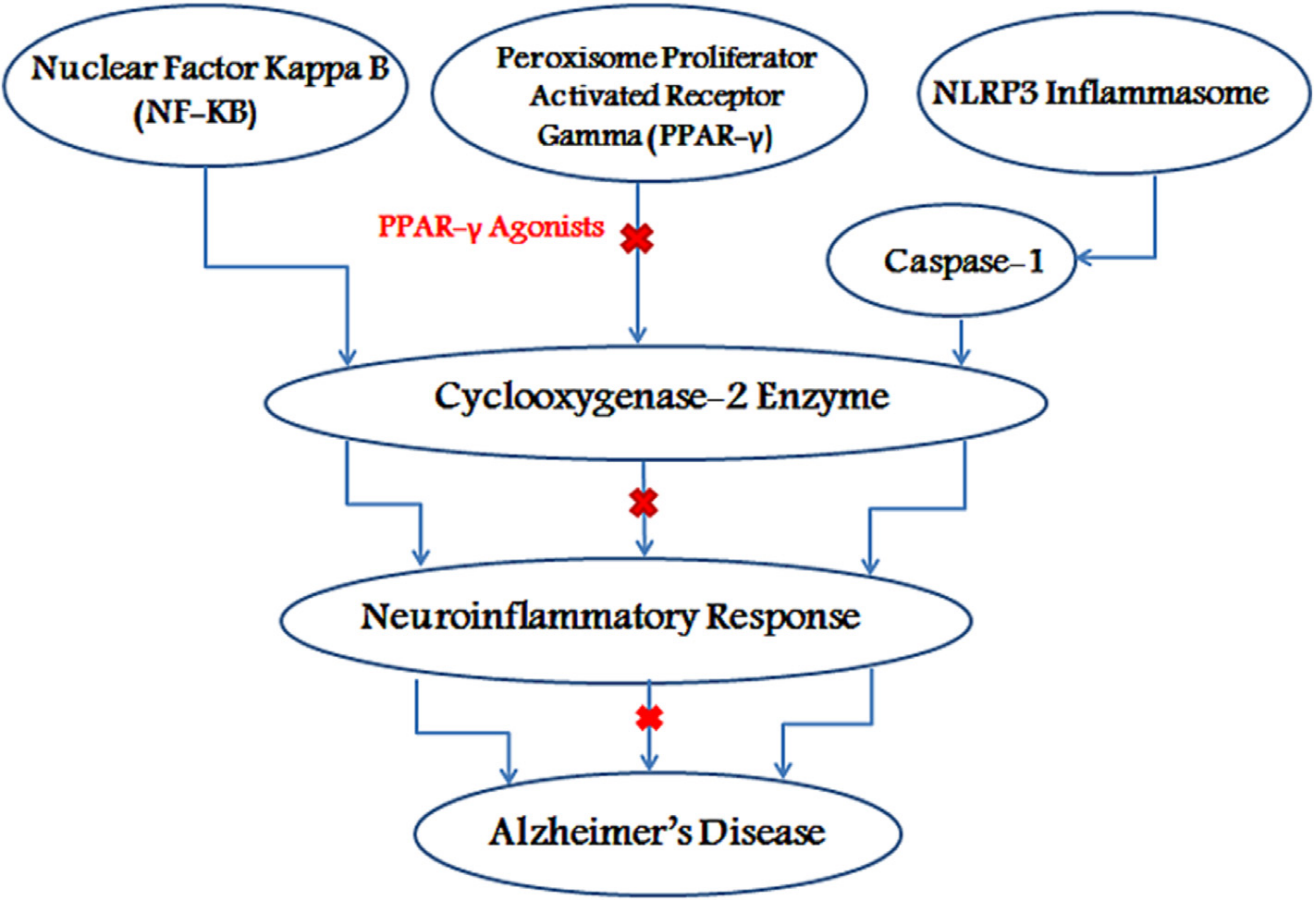
Nuclear Factor Kappa B (NF-kB) activation increases COX-2 expression in microglia which results in increased neuroinflammatory response, while PPAR-γ activation via PPAR-γ agonists reduces COX-2 expression and decreases the inflammatory response, the NLRP3 inflammasome activates caspase-1, which in turn leads to induced COX-2 expression and eventually AD.

One more protein that has been studied in relation to COX-2 in AD is Nuclear Factor Kappa B (NF-kB). It is a transcription factor that is activated by pro-inflammatory cytokines and is involved in the regulation of the expression of genes related to inflammation, including COX-2. Studies have shown that the activation of NF-kB may contribute to the development of AD by increasing the expression of COX-2 in microglia, leading to increased production of pro-inflammatory molecules that exacerbate the neuroinflammatory response in AD (Fig. 2) (Amor et al., 2010, Shabab et al., 2017).

## 5 COX-2 and peroxisome Proliferator-Activated receptor gamma (PPAR-γ)

Another protein that has been studied in relation to COX-2 in AD is Peroxisome Proliferator-Activated Receptor Gamma (PPAR-γ). PPAR-γ is a nuclear receptor that plays a role in the regulation of inflammation and has been shown to have anti-inflammatory effects through the inhibition of COX-2 expression (Du et al., 2011). Studies have suggested that PPAR-γ agonists may have therapeutic potential in AD by reducing the expression of COX-2 and decreasing the neuroinflammatory response (Chen et al., 2012, Bordet et al., 2006; Kapadia et al., 2008; Lastra et al., 2004).

Epidemiological studies indicate that patients with a history using non-steroidal anti-inflammatory drugs (NSAID, COX1/2 inhibitor, and PPARγ agonist) have decreased risk for neurodegeneration, such as AD (Fig. 2).

## 6 COX-2 and the NLRP3 inflammasome

The NLRP3 inflammasome is a protein complex that plays a key role in the inflammatory response (Im and Ammit, 2014). It is activated by a wide range of different stimuli, including bacterial and viral infections (Swanson et al., 2019), as well as damage to cells and the accumulation of certain proteins or molecules (Huang et al., 2021). Once activated, the NLRP3 inflammasome activates caspase-1, which in turn leads to the production of the pro-inflammatory cytokines IL-1β and IL-18. These cytokines play a critical role in the body's immune response to infection and injury, (Swanson et al., 2019) but their overproduction has been linked to a number of diseases, including Alzheimer's disease, obesity, and type 2 diabetes (Tan et al., 2013).

There is a relationship between the NLRP3 inflammasome and COX-2 (cyclooxygenase-2) in the context of inflammation. COX-2 is an enzyme that catalyzes the production of prostaglandins, which are important mediators of inflammation. Studies have shown that the activation of the NLRP3 inflammasome can lead to the increased expression of COX-2, which in turn leads to an increase in prostaglandin production. This results in an amplification of the inflammatory response (Hua et al., 2015).

Furthermore, inhibition of COX-2 has been shown to reduce the activation of NLRP3 inflammasome and its downstream cytokines. Some studies have reported that non-steroidal anti-inflammatory drugs (NSAIDs) such as flufenamic acid, meclofenamic acid, mefenamic acid, ibuprofen and celecoxib (Hua et al., 2015) can reduce the activation of the inflammasome and the production of its downstream cytokines, which may be beneficial in conditions characterized by chronic inflammation (Daniels et al., 2016, Liu et al., 2020).

Celecoxib, on the other hand, is a non-steroidal anti-inflammatory drug (NSAID) that specifically targets the COX-2 enzyme.

In other words, there is a relationship between the NLRP3 inflammasome, COX-2, and Alzheimer's disease (AD). The NLRP3 inflammasome and its downstream cytokines are activated in the brains of AD patients, and that this activation may contribute to the inflammation and neuronal damage observed in AD. The inhibition of COX-2 can reduce the activation of the NLRP3 inflammasome and the production of its downstream cytokines. This suggests that targeting the NLRP3 inflammasome and COX-2 may be a potential therapeutic plan for treating neuroinflammation and slowing the progression of AD.

In summary, the COX-2 enzyme plays a significant role in the pathogenesis of Alzheimer's disease. It has been shown to be involved in learning and memory functions in the hippocampus, which is one of the main areas affected in AD. Overexpression of COX-2 has been correlated with impairment of spatial memory and is expressed under normal physiological conditions in the brain. However, it is also induced by inflammatory stimuli and is found to be elevated in microglial and astroglial cells. The activation of these cells leads to the release of pro-inflammatory cytokines, which contribute to the neuroinflammatory response seen in AD. Studies have also shown that COX-2 expression varies according to the different stages of AD, with high levels being present in the early stages but decreasing as the disease progresses.

## 7 COX-2 and acetylcholine

Acetylcholine (ACh) is a neurotransmmiter that plays a vital role in many aspects of brain function such as learning and memory (Chen et al., 2022, Liu et al., 2017), Ach is synthesized at nerve terminals from choline (Ch) and acetyl coenzyme A (Acetyl-CoA) by the action of the enzyme choline acetyltransferase (Liu et al., 2017) and hydrolyzed rapidly by the enzyme acetylcholinesterase (AChE) (Beckmann et al., 2013). This chemical messenger alters neuronal excitability (Wang et al., 2021b, Wang et al., 2021a), regulates the synaptic activity (Colangelo et al., 2019) and helps to send messages between some neurons (Sam and Bordoni, 2022). ACh deficiency has been linked to AD pathology. Recent studies showed that low levels of ACh as a result of gradual death of cholinergic neurons led to severe loss of cognitive function in affected patients (Chen et al., 2022). As well as, significant reduction in both (Stepanichev et al., 2017) and AChE activity was also observed in the brains of AD patients (Campanari et al., 2016), hence, the cholinergic hypothesis has arisen (Chen et al., 2022), and number of acetylcholinesterase inhibitors (AChEIs) such as donepezil, galantamine, rivastigmine are used as therapeutic agents to increase ACh concentrations in the brain and improve memory (Vecchio et al., 2021).

A study found that AChEIs not only increase brain ACh levels, but can also reduce the inflammatory response which plays a pivotal role in AD by lowering the secretion of pro-inflammatory molecules like PGE2 and TXB2 and the expression of both enzymes COX-1 and COX-2. Which means that this class of drugs have anti-inflammatory properties (Goschorska et al., 2018).

Previous studies have shown that acetylcholine can modulate the activity of the COX-2 enzyme. In particular, research has found that acetylcholine can inhibit the activity of COX-2, thereby reducing the production of prostaglandins and the associated inflammation. This mechanism may be involved in the anti-inflammatory effects of acetylcholine, which have been observed in various conditions such as osteoarthritis and Alzheimer's disease (Goschorska et al., 2018). Additionally, studies suggest that COX-2 inhibitors may enhance the effects of acetylcholine by increasing its availability in the brain (Scali et al., 2003).

**Fig. 3:**
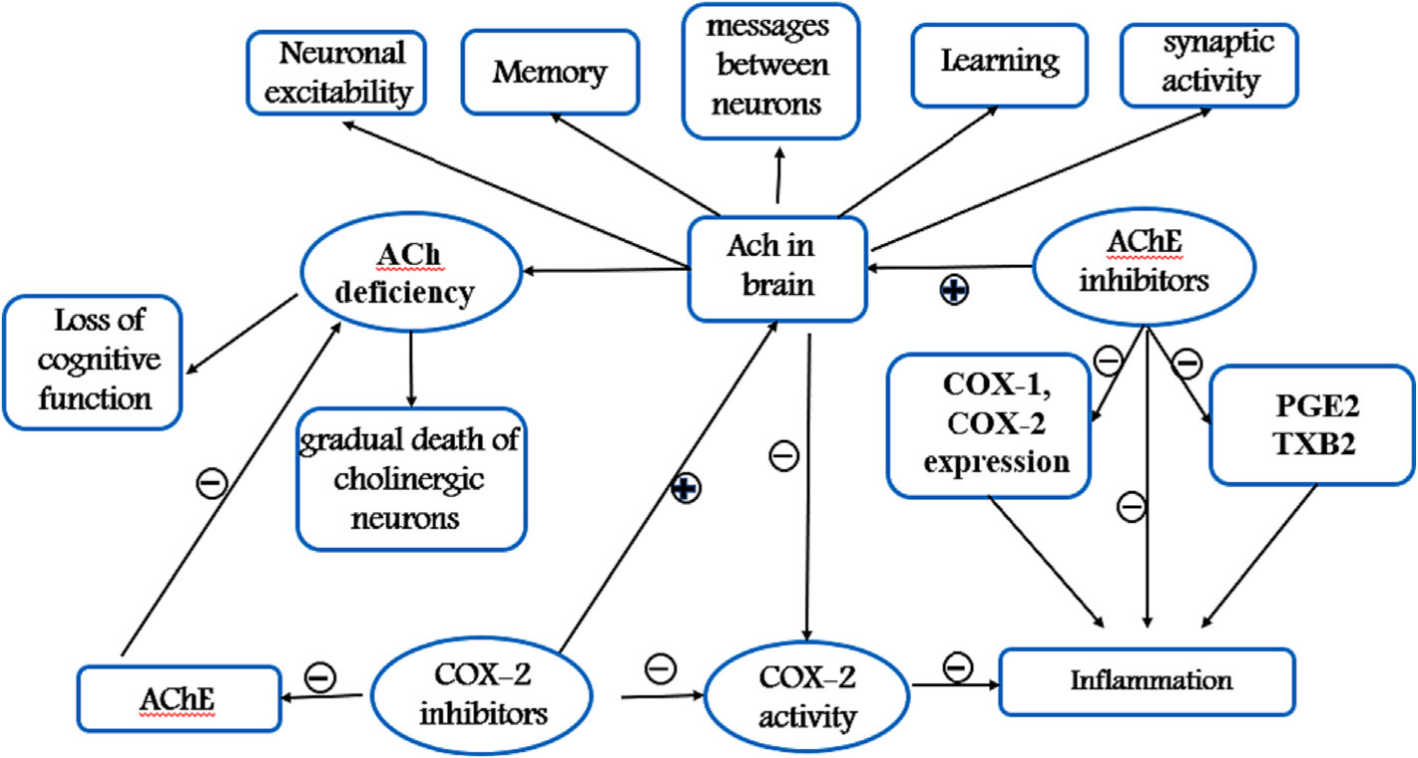
Summary of the relationship among ACh, COX-2, Inflammation and AD pathology.

Donepezil is a medication used in the treatment of Alzheimer's disease (AD) and is classified as an acetylcholinesterase inhibitor (AChEI). Both in vitro and in vivo studies have shown that Donepezil can reduce the expression of COX-2 and microglial activation in AD brains. This suggests that the drug may have anti-inflammatory effects in addition to its primary mechanism of action as an AChEI (Kim et al., 2021). On the other hand, recent studies have shown that celecoxib can also inhibit AChE (Gaur & Kumar, 2011) and this has been demonstrated through molecular docking studies, which showed an interaction between celecoxib and amino acid residues at the active site of AChE enzyme. This suggests that celecoxib may have dual action, as it acts as an AChE inhibitor and a COX-2 inhibitor (Pohanka, 2017). By targeting both COX-2 and AChE, celecoxib may have the potential to provide a more comprehensive approach to treating the disease. The dual action of celecoxib may have implications for the treatment of AD. The relationship between ACh, COX-2, and inflammation as well as the pathology of AD has summarized in Fig. 3.

## 8 COX-2 and beta amyloid

The amyloid hypothesis describes beta amyloid (Aβ) deposits in the brain to be one of the main pathological hallmarks in AD (Soria Lopez et al., 2019).

Aβ is a peptide (Dinkel et al., 2020) that is derived from the amyloid precursor protein (APP) through sequential cleavage by β-secretase and γ-secretase (Chen et al., 2017). The resulting peptide can vary in length, with the most common forms being Aβ40 and Aβ42, which are 40 and 42 amino acids in length, respectively. These peptides can aggregate to form amyloid plaques, which are a mark of Alzheimer's disease (Hampel et al., 2021, Dehury et al., 2019).

APP undergoes sequential cleavage by β-secretase and γ-secretase along two distinct pathways (Chen et al., 2017). The amyloidogenic pathway that leads to the formation of Amyloid Beta (Aβ) by β- and γ-secretases. APP is first cleaved by β-secretase (BACE1) to generate soluble APPβ (sAPPβ) and the membrane-bound C99 (Hampel et al., 2021) which is cleaved by γ-secretase leading to the secretion of Aβ (Dehury et al., 2019).

The second one is the non-amyloidogenic pathway in which APP is cleaved by α-secretase resulting in soluble APPα (sAPPα) (Wang and Qu, 2016) and a membrane-bound C83 which is also cleaved by γ-secretase (Hur, 2022). An imbalance in the levels of Aβ can lead to the Aβ aggregation of the peptide into amyloid plaques. These plaques are found in the brain, and can cause neurotoxicity, brain inflammation (Soria Lopez et al., 2019) and disrupt nerve cell connections https://www.nia.nih.gov/health/what-happens-brain-alzheimers-disease, leading to cognitive decline and memory loss.

It's important to note that the exact mechanisms by which amyloid plaques cause neurotoxicity are still under investigation and the relationship between Aβ aggregation and the onset of Alzheimer's disease is complex and still not fully understood. Current research is focused on understanding the underlying causes and mechanisms of Aβ aggregation and developing potential therapies to target this process in order to slow or stop the progression of the disease.

A number of studies in transgenic mice have found that overexpression of the COX-2 enzyme leads to accumulation of beta-amyloid (Aβ) in amyloid plaques in the cerebellum, a region of the brain involved in motor coordination and cognitive function. This accumulation of Aβ plaques in the cerebellum has been associated with cognitive deficits in these mice (Guan et al., 2019, Guan and Wang, 2019, Xiang et al., 2002).

Additionally, a number of studies have found increased COX-2 immunoreactivity in the brains of Alzheimer's disease (AD) patients, and that this increased COX-2 immunoreactivity is related to the number of amyloid plaques in the brain (Hoozemans et al., 2008, Kadoyama et al., 2002, Hwang et al., 2002).

Studies have shown that hippocampal COX-2 expression may have an effect on the cleavage of the amyloid precursor protein (APP) by influencing gamma-secretase, an enzyme involved in the production of beta-amyloid (Aβ) peptides (Xiang et al., 2002). COX-2 overexpression in the hippocampus of transgenic mice led to an increase in the production of Aβ42, which tends to self-assemble and form aggregates (Soria Lopez et al., 2019, Xiang et al., 2002, Kadoyama et al., 2002, Pogue et al., 2015).

Another study by Weggen et al found that COX-2 inhibition reduced the production of Aβ42 and the formation of amyloid plaques in the hippocampus of transgenic mice (Weggen et al., 2001).

In addition to this, metabolic products of COX-2 such as PGE2 have been suggested to be involved in the pathogenesis of AD via increasing Aβ aggregation. Elevated levels of PGE2 in the brains of transgenic mice have been shown to affect both beta- and gamma-secretase, leading to an increase in the production of Aβ peptides (Guan et al., 2019).

Thromboxane A2 (TXA2), a metabolic product of COX-2, has also been suggested to exacerbate Alzheimer's disease (AD) neuropathology by inducing expression of the APP and beta-amyloid (Aβ) secretion (Biringer, 2019). These findings suggest that COX-2 and its metabolic products can play a critical role in the production and aggregation of Aβ in the brain, and may contribute to the development and progression of Alzheimer's disease (Guan et al., 2019, Xiang et al., 2002).

Studies have shown that Non-Steroidal Anti-Inflammatory Drugs (NSAIDs) have the ability to decrease the levels of Aβ in the brain and inhibit Aβ aggregations.

Data from epidemiological, in silico, and in vivo studies have explained the importance of using NSAIDs to decrease the levels of Aβ in the brain. Molecular docking studies have predicted the interaction between NSAIDs and Aβ fibrils, and it has been shown that different NSAIDs have different affinities towards Aβ fibrils. For example, naproxen has been shown to have a higher affinity towards Aβ fibrils than ibuprofen (Azam et al., 2017).

In vivo studies have demonstrated that NSAIDs have an effect on gamma-secretase activity and can decrease the amyloid burden by modulating this enzyme. For example, meclofenamic acid and enantiomers of flurbiprofen have been shown to decrease the amyloid burden (Tyagi et al., 2021; Eriksen et al., 2003).

In vitro study has shown that flufenamic acid, mefenamic acid, and meclofenamic acid inhibit IL-1β secretion more effectively than ibuprofen or celecoxib, and slow cognitive deterioration caused by Aβ in mice. Treatment with diclofenac also reduced Aβ burden (Stuve et al., 2020).

In addition to their inhibition of COX enzymes, NSAIDs activate peroxisome proliferator-activated receptor gamma (PPARγ) which induces a clearance mechanism for the Aβ depositions (Sastre et al., 2008, Dhapola et al., 2021). Some NSAIDs have been reported to target APP which has an important role in AD pathology. For example, aspirin, indomethacin and flurbiprofen have been shown to decrease the levels of Aβ via reducing the activity of NFkβ, which is an important mediator of inflammation. (Jaturapatporn et al., 2012, Kinney et al., 2018).

Overall, the relationship between COX-2 and Aβ is complex and COX-2 inhibitors may have potential as a therapeutic drug for Alzheimer's disease.

## 9 COX-2 and tau protein

Tau is a protein that plays a vital role in the stability and modulation of internal microtubules in the brain (Brotzakis et al., 2021). Microtubules are a component of the cytoskeleton, which are essential for many fundamental cellular processes such as regulating cell growth (Muralidar et al., 2020). The phosphorylation of tau protein is determined by a balance in the activities of both tau kinases and tau phosphatases. However, in the brains of people with Alzheimer's disease (AD), tau becomes hyperphosphorylated, which causes it to detach from microtubules and form fibers that accumulate as neurofibrillary tangles (NFTs). These NFTs are a fundamental neuropathological hallmark of AD (Wang et al., 2017, Ghosh et al., 2013, Gorantla et al., 2019**).**

There is a significant amount of evidence demonstrating the critical role of COX-2 in the phosphorylation of tau protein. A study in transgenic mice has shown that metabolic products of COX-2, like PGF2α, and elevated PGI2 (Wang et al., 2017), induce tau hyperphosphorylation leading to deficits in cognitive function and AD development and progression (Guan and Wang, 2019).

It is believed that overexpression of cytokines such as TNF-α, IL-1β, IL-18, and IL-6 accelerate the phosphorylation of tau, leading to the development of NFTs and impairing the learning ability of mice by inducing the expression of the COX-2 enzyme (Wang et al., 2017, Ghosh et al., 2013).

Additionally, 15-Deoxy-Δ-12,14-prostaglandin J2 (15d-PGJ2), which is synthesized from PGD2, may contribute to the pathogenesis of AD via exacerbating tau pathology (Guan and Wang, 2019).

In vivo studies have implicated the role of COX-2 expression in reducing the glycosylation of tau protein, which is a post-translational modification leading to the phosphorylation of tau (Guan and Wang, 2019).

Results indicate that calcium ions are associated with tau phosphorylation via inducing COX-2 expression. There is also an extent of colocalization between COX-2 and phosphorylated tau protein in both the brainstem and spinal cord of transgenic mice with AD, as detected by immunofluorescence (Guan and Wang, 2019, Cao et al., 2019).

A study in patients with Fukuyama-type congenital muscular dystrophy demonstrated that COX-2 overexpression induces the formation of NFTs.

Taken together, these findings demonstrate that COX-2 induces the phosphorylation of tau, raising the question as to whether non-steroidal anti-inflammatory drugs (NSAIDs) are useful in decreasing hyperphosphorylated tau protein. Many studies have revealed that treatment with rofecoxib, an inhibitor of COX-2 (Guan and Wang, 2019) reduces tau hyperphosphorylation. Other NSAIDs, including ibuprofen, have been shown to reduce memory impairment via inhibiting COX-2 and lowering the levels of phosphorylated tau (Guan and Wang, 2019, Wang et al., 2017**)**.

## 10 COX-2 and GSK3β

Glycogen Synthase Kinase 3 Beta (GSK3β) is a protein kinase that plays a critical role in the phosphorylation of tau protein (Zhou et al., 2022). This enzyme plays an important role in several aspects of neuronal function, such as neuronal development (Luo, 2012). GSK3β is one of the main kinases that phosphorylates tau protein and has been linked to the pathogenesis of Alzheimer's disease (AD).

The overactivity of GSK3β increases the hyperphosphorylation of tau, disruption of synaptic plasticity (Zhou et al., 2022; Sayas, 2021) and can contribute to the accumulation of Aβ via affecting presenilin-1 (PS1) (D’Mello, 2021).

This dysregulation of GSK3β has been linked to the pathogenesis of AD (Sayas, 2021). For example, a study in transgenic mice found that overexpression of GSK3β leads to tau hyperphosphorylation and cognitive impairment (Rodríguez-Matellán et al., 2020).

It is thought that the relationship between GSK3β and COX-2 is a significant contributor to the pathogenesis of Alzheimer's disease (AD). Studies have reported that GSK3β plays a central role in the inflammatory process (Jope et al., 2007).

In vitro and in vivo studies demonstrated that GSK3β is implicated in the secretion of inflammatory molecules like PGE2 (Noma et al., 2016), cytokines including IL-1, IL-6 and TNF-α (Lauretti et al., 2020) and activation of microglial cells (D’Mello, 2021, Eskandarzadeh et al., 2021).

Interestingly, there is a link between COX-2 and GSK3β. Studies have shown that COX-2 can regulate GSK3β activity, and that inhibition of COX-2 can reduce GSK3β activity and tau phosphorylation. For example, a study found that COX-2 inhibition reduces tau phosphorylation by decreasing GSK3β activity in a mouse model of AD. It has also been reported that 15d-PGJ2, a metabolite of COX-2, can activate GSK3β and exacerbate tau pathology.

The inhibition of GSK3 significantly reduces the expression of both COX-2 and mPGES-1 (Eskandarzadeh et al., 2021), which are enzymes involved in the inflammatory process.

One study found that inhibition of GSK3β reduced COX-2 expression and PGE2 production in a mouse model of AD (Czapka et al., 2020, Takadera and Ohyashiki, 2006). Another study found that treatment with a GSK3 inhibitor called SB216763 led to a reduction in the expression of COX-2 (Oliveira et al., 2012). These findings suggest that GSK3 inhibitors have anti-inflammatory effects (Jope et al., 2007).

Therefore, GSK3β inhibitors would be promising in the treatment of neurodegenerative disorders where neuroinflammation plays a key role (Takadera and Ohyashiki, 2006).

A growing body of research has been dedicated to studying the anti-cancer properties of anti-inflammatory drugs such as celecoxib, which has been found to inhibit GSK3 (Chen et al., 2011).

Similarly, another study focused on naproxen for its inhibitory effects on GSK3 to help in the management of diabetes and obesity. Naproxen has been found to inhibit GSK3 in adipose tissue, leading to improved insulin sensitivity and reduced inflammation (Motawi et al., 2013).

Flurbiprofen and ibuprofen are also among the NSAIDs that have been found to inhibit the activity of GSK3β (Fu et al., 2012, Greenspan and Madigan, 2011). A study was designed to elucidate the effects of tolfenamic acid on GSK3β activity and found that GSK3β was inhibited by tolfenamic acid, a member of the fenamate group, leading to a decrease in hyperphosphorylated tau protein levels (Fu et al., 2012, Greenspan and Madigan, 2011, Zhang et al., 2020, Ahmed et al., 2018).

GSK3β plays a central role in the inflammatory process and has been linked to the pathogenesis of AD. Studies have also shown that GSK3β is related to COX-2, and that inhibition of GSK3β reduces the expression of COX-2, suggesting that GSK3 inhibitors have anti-inflammatory effects, and could be promising in the treatment of neurodegenerative disorders where neuroinflammation is a main factor.

In conclusion, these findings provide strong evidence that a number of NSAIDs could be useful by blocking GSK3 enzyme leading to an attenuation in the levels of phosphorylated tau protein, resulting in a reduced risk of developing cognitive impairment (Bradbury, 2004).

## 11 COX-2 and CDK5

Cyclin-dependent kinase 5 (CDK5) is a protein kinase that belongs to the CDK family (Allnutt et al., 2020). Unlike other CDK proteins, which need to bind to cyclins to become active, CDK5 is activated upon binding with its specific activators p35 or p39 (Liu et al., 2016). CDK5 plays a critical role in many essential functions such as the normal development of the central nervous system (CNS), neuronal migration, synaptic function (Allnutt et al., 2020) and memory (D’Mello, 2021). However, in addition to these physiological roles, CDK5 is also suggested to be involved in the pathogenesis of many neurodegenerative diseases including Alzheimer's disease (AD), as it is a major tau protein kinase.

In vitro studies have demonstrated that overactivity of CDK5 leads to hyperphosphorylation of tau and increased formation of neurofibrillary tangles (NFTs) (Noble et al., 2003). CDK5 has also been reported to contribute to the inflammatory process (Pfänder et al., 2021). It has a role in the secretion of inflammatory cytokines (D’Mello, 2021). A study confirmed that both GSK3β and CDK5 have roles in neurodegenerative diseases where neuroinflammation (Reinhardt et al., 2019) takes a role. Inflammatory mediators including IL-6 and TNF-α have been shown to trigger the activation of CDK5 (Wilkaniec et al., 2018).

A previous study reported that roscovitine, a CDK5 inhibitor, showed anti-inflammatory properties and reduced the high levels of COX-2 (Wilkaniec et al., 2018), indicating that this drug could have beneficial effects in neuroinflammatory-mediated degeneration (Pfänder et al., 2021).

A study has been dedicated to studying the role of NSAIDs in preventing cognitive function disorder through many pathways and it showed that dexibuprofen could decrease the inflammatory stimuli via affecting the CDK5 pathway and diminishing the phosphorylation of tau protein (Ettcheto et al., 2017). This suggests that CDK5 inhibitors could be a promising approach in the treatment of neurodegenerative disorders such as Alzheimer's disease, where tau phosphorylation and neuroinflammation play critical roles in the pathology.

**Fig. 4:**
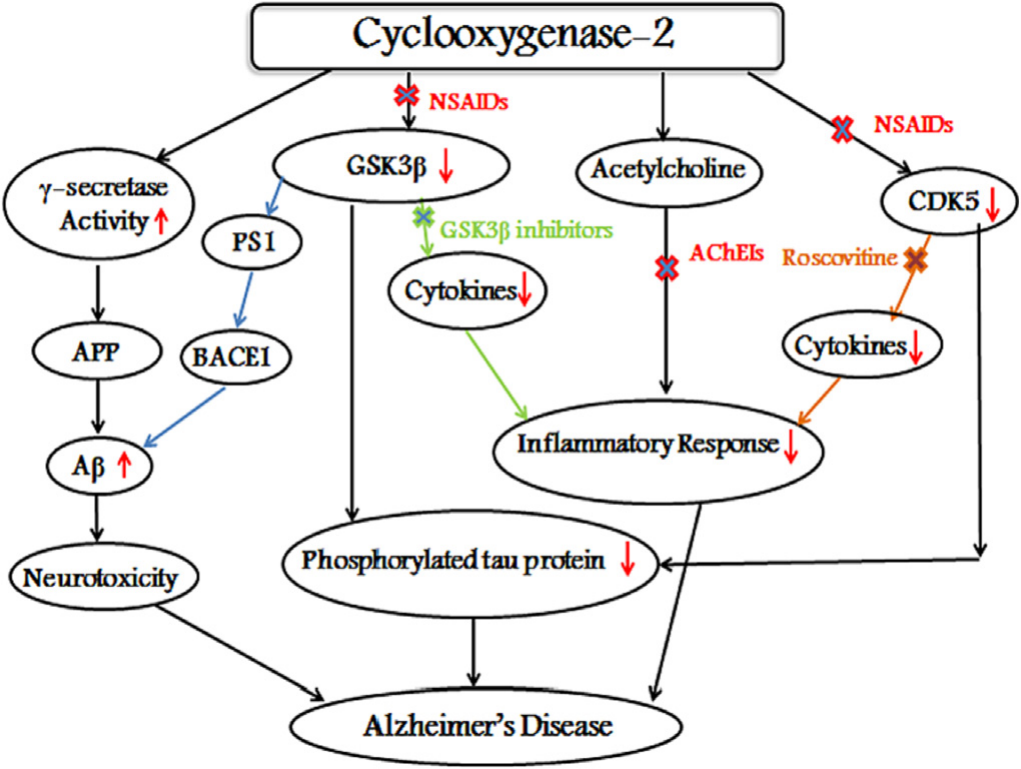
Involvement of COX-2 enzyme in AD. COX-2 induces γ-secretase activity which in turn cleaves APP leading to the neurotoxic Aß generation which is associated with AD, while the inhibition of Gsk3ß and Cdk5 by a number of NSAIDs reduces the expression of COX-2 leading to an attenuation in the levels of phosphorylated tau protein. ACHEIs could reduce the inflammatory response via increasing ACh concentrations in the brain.

As a result, the development of CDK5 inhibitors offers a new approach in the treatment of brain inflammation (Wilkaniec et al., 2018). Fig. 4 shows the relationship between COX-2 and Alzheimer's related proteins.

## 12 Role of NSAIDs in the management of AD

Neuroinflammation has been proposed as an underlying mechanism that is implicated in a number of neurodegenerative diseases including Alzheimer's disease (AD) (Spagnuolo et al., 2018). Therefore, the management of AD with non-steroidal anti-inflammatory drugs (NSAIDs) has received increasing attention (Aisen, 2002). NSAIDs have been hypothesized to prevent the progression and development of AD (Meyer et al., 2019).

Several studies have demonstrated that long-term use of NSAIDs is associated with a reduced risk of AD and may protect against it. A number of epidemiological studies have shown good results and promising potential in this field. These studies have examined the association between the use of this class of medications and the progression of AD.

The Baltimore Longitudinal Study of Aging found that the risk of AD decreased for people who used NSAIDs for more than 2 years (Ozben and Ozben, 2019). Results from other studies have also suggested that NSAIDs may have protective effects against AD. For example, the Rotterdam Study reported that NSAIDs have effects on dementia risk and could reduce it (In ’t Veld et al., 1998). Another study found that a high-dose of aspirin may lower the incidence of AD (Nilsson et al., 2003). While the key finding of Jorda's study was that the administration of low doses of aspirin might be beneficial in AD patients (Jorda et al., 2020).

On the other hand, a study showed that aspirin at a low-dose could slow down the rate of cognitive impairment (Nguyen et al., 2022). It was also found that NSAIDs have a protective role and appeared to contribute to regulatory processes of glutamate function and enhance synaptic function (Imbimbo, 2009). A study found that indomethacin at a dose of 100–150 mg per day showed neuroprotective effects in patients with AD (P. R et al., 2021). It has been shown that ibuprofen also has beneficial effects in the management of AD and improves memory deficits (Zamanian-Azodi et al., 2019).

As mentioned before, during the progression of AD, microglial cells contribute to the neuroinflammatory process. Therefore, a number of studies have shown that some NSAIDs suppress the activation of microglia (Gunaydin and Bilge, 2018, Imbimbo, 2009). For example, a study found that celecoxib, an NSAID that selectively targets COX-2, reduced the activation of microglia in a mouse model of AD. Another study found that ibuprofen suppressed the activation of microglia and reduced the levels of phosphorylated tau protein.

But there is controversial data from preclinical studies which focused on the effects of NSAIDs especially selective COX-2 inhibitors in AD and found that celecoxib has failed to show cognitive amelioration in the brains of AD patients (Sánchez-Sarasúa et al., 2022).

While there is evidence that suggests that non-steroidal anti-inflammatory drugs (NSAIDs) may have protective effects against Alzheimer's disease (AD), there are also studies that have produced conflicting results. Some preclinical studies have focused on the effects of NSAIDs, particularly selective COX-2 inhibitors, in AD and found that they have failed to show cognitive improvement in the brains of AD patients. For example, a study found that celecoxib did not show cognitive amelioration in AD patients (Tyagi et al., 2020, Rivers-Auty et al., 2020; Fu et al., 2018).

Additionally, clinical studies have provided evidence that the use of NSAIDs has been disappointing in the management of AD and did not slow down the severity of dementia (Tyagi et al., 2020; P. R et al., 2021; Fu et al., 2018).

For example, treatment with ibuprofen at 400 mg had no significant cognitive benefit (Elmaleh et al. 2019), and naproxen at a dose of 220 mg was not effective in preventing the progression of pre-symptomatic AD (Benito-León et al., 2019).

Furthermore, some studies have suggested that certain NSAIDs may even worsen the disease. For example, studies evaluating whether rofecoxib could have beneficial effects in the management of AD have been negative and have shown that rofecoxib has failed and may have been associated with aggravation of AD (Maccioni et al., 2020, Cai et al., 2019).

These results highlight the potential explanations for the failure of NSAIDs and selective COX-2 inhibitors against AD.

It was suggested that NSAIDs, if started early enough, could have a preventive role (Zimmer et al., 2014) the failure of NSAIDs and selective COX-2 inhibitors against AD may be due to the timing and progression of AD and the type of NSAID used (Biringer, 2019). Therefore, it is believed that NSAIDs may be a promising novel therapeutic approach for the management of neurodegenerative diseases including AD, but further research and studies are needed to fully understand their effects and to develop new NSAIDs.

It is important to note that the results of preclinical and clinical studies investigating the use of COX-2 inhibitors in AD are mixed, and the potential benefits and risks of these drugs in the treatment of AD is still under investigation. It's also important to note that the studies that showed the benefits of COX-2 inhibitors in AD are still preclinical studies and more large-scale clinical trials are needed to confirm these results.

In conclusion, several studies have provided promising evidence that NSAIDs may have protective effects against AD. These studies have shown that long-term use of NSAIDs is associated with a reduced risk of AD and that some NSAIDs can reduce the levels of phosphorylated tau protein, improve cognitive function, and suppress the activation of microglia.

## 13 Conclusion

In summary, the purpose of this review was to examine the potential role of cyclooxygenase enzymes, specifically COX-2, in the development and progression of Alzheimer's disease (AD). It highlighted evidence that suggests that COX-2 may be an important therapeutic target in AD as it contributes to neuronal activities and is upregulated in the brains of people with AD. It also discussed the association between the elevated expression of COX-2 and other key proteins that contribute to the development of AD.

The review also discussed the potential use of non-steroidal anti-inflammatory drugs (NSAIDs) as a treatment for AD. As NSAIDs are known inhibitors of COX enzymes, they may have beneficial effects by interfering with the chronic inflammatory process that is thought to be involved in AD. However, the review also acknowledged that there are mixed results in studies examining the effectiveness of NSAIDs in treating AD, and that more research is needed in this area.

Although, there are currently no FDA-approved medications specifically designed to target COX-2 in the treatment of Alzheimer's disease. the review suggests that COX-2 may be a promising target for the treatment of AD, and that NSAIDs may have potential as a treatment for AD. However, it also emphasizes the need for further research and studies to fully understand the mechanisms by which COX-2 and NSAIDs may be involved in the development and progression of AD and to identify new therapeutic strategies for the management of AD.

## Author contribution

All authors contributed to the conception of this review and its design. Nathalie Moussa planned and structured the review. Both authors created the first draft and all the illustrations. In previous versions of the manuscript, all authors provided their comments. The final manuscript was read and approved by all authors.

## Declaration of Competing Interest

The authors declare that they have no known competing financial interests or personal relationships that could have appeared to influence the work reported in this paper.
